# A Case of Rupture of the Stomach, Accompanied by Violent Spasm, and Arising without Any Evident Cause

**Published:** 1830-06

**Authors:** C.J. Roberts

**Affiliations:** Physician to the General Dispensary, Aldersgate street.


### THE LONDON
### Medical and Physical Journal.

N° 376, VOL. LXIII.] JUNE 1830. [N° 48, *New Series*.

For many fortunate discoveries in medicine, and for the detection of numerous errors, the world is indebted to the rapid circulation of Monthly Journals; and there never existed any work to which the Faculty, in Europe and America, were under deeper obligations than to the *Medical and Physical Journal of London*, now forming a long but an invaluable series.—*Rush.*

### ORIGINAL PAPERS, AND CASES,

### OBTAINED FROM PUBLIC INSTITUTIONS AND OTHER AUTHENTIC SOURCES.

### RUPTURE OF THE STOMACH.

*A Case of Rupture of the Stomach, accompanied by violent Spasm, and arising without any evident Cause.* By C. J. ROBERTS, M.D., Physician to the General Dispensary, Aldersgate street.

JOHN DUNN, æt. four, was brought to my house, complaining of pain in the abdomen, which was swollen and tense, with great listlessness and languor: his eyes were dull and heavy, countenance pallid, with a foul tongue and considerable emaciation. He had a short irritable cough, pain in the right side, and some dyspnœa. His mother informed me that, some time previous, he had discharged a lumbricus teres, of coniderable size, from the stomach by vomiting. These complaints continuing to increase, I was requested to visit him at home: I attended him for rather more than a fortnight, during which time I freely purged him, and gave him some medicine to alleviate the cough, which was without expectoration and very troublesome, and applied a blister to the right side, which rose well and relieved the pain. The evacuations from the bowels were dark-coloured and fœtid, altogether demonstrating a very depraved state of the stomach and bowels. He, however, slowly recovered, and I left him, although weak, yet without any apparent disease.

About a week from my having taken leave of him, I was hastily summoned to see him, as he was stated to be in a fit, and without the least expectation of recovery, unless immediate relief could be procured by medical aid. Being from home, I did not see him so promptly as I could have wished; but, instantly on my return, I visited him, and found him lying on his left side: the whole of the muscles of the right side of the body, from the face to the toes, were in a state of violent spasmodic action; the teeth were firmly clenched, excepting at intervals when they ground against each other, and all attempts to separate them were vain. I ordered him immediately into a warm bath, and prescribed some antispasmodic medicine, but he was unable to swallow. The bath afforded some trifling, though temporary relief; and he died in about five hours and a half from the first appearance of the spasms.

His mother could not give the slightest reason for the occurrence of the fit, as he had appeared more than commonly cheerful, and had eaten more heartily than usual, although by no means voraciously, about an hour and a half previous to his being attacked.

Permission was obtained the next day to open the body, which was done with considerable care and attention by my friends, Messrs. Eden and James.

There were extensive and old adhesions of the right lung to the pleura costalis, but the left lung did not afford the slightest traces of inflammation. The heart was rather larger and more flabby than usual, but exhibited no other trace of disease. There was some slight accumulation of water in the pericardium.

The general appearance of the intestines was healthy. On lifting the omentum, and passing the hand behind the stomach, a quantity of chyme, and other contents of the stomach, was found lying in the abdominal cavity: this viscus was then very carefully removed and examined, when a tear or rent of considerable size was discovered at the cardiac extremity, which included nearly the whole of that end of the stomach. It was most carefully slit up, and every part scrutinised to endeavour to discover the presence of either inflammatory action, or any ulceration or attenuation of the coats of that organ; but none such were discoverable, and we were therefore unable to assign any reason for the rupture. The mesenteric glands were rather enlarged and vascular, more especially one situated near the head of the colon, which was nearly as large as a sparrow's egg.

It is much to be regretted that we were not allowed to open the head.

After this rupture of the stomach was discovered, I questioned the mother very particularly, whether he had fallen from the bed, or had received a blow on the stomach soon after eating, or had been leaning over the side of the high crib in which he was lying, so as to have pressed upon the loaded viscus; but she denied any such circumstance having occurred, and left us as much in the dark as before as to the probable cause of the accident.

31, *Bridge street; May* 1830.

---

### LUXATION OF THE CERVICAL VERTEBRÆ.

### *Case of Luxation of the Cervical Vertebræ.*

By Dr. Ehrlich.*

